# Basilar Membrane Responses to Tones and Tone Complexes: Nonlinear Effects of Stimulus Intensity

**DOI:** 10.1007/s10162-012-0345-0

**Published:** 2012-08-31

**Authors:** Corstiaen P. C. Versteegh, Marcel van der Heijden

**Affiliations:** Department of Neuroscience, Erasmus MC, P.O. Box 2040, 3000 CA Rotterdam, The Netherlands

**Keywords:** cochlear mechanics, laser interferometry, quasilinear filtering, automatic gain control

## Abstract

The mammalian inner ear combines spectral analysis of sound with multiband dynamic compression. Cochlear mechanics has mainly been studied using single-tone and tone-pair stimulation. Most natural sounds, however, have wideband spectra. Because the cochlea is strongly nonlinear, wideband responses cannot be predicted by simply adding single-tone responses. We measured responses of the gerbil basilar membrane to single-tone and wideband stimuli and compared them, while focusing on nonlinear aspects of the response. In agreement with previous work, we found that frequency selectivity and its dependence on stimulus intensity were very similar between single-tone and wideband responses. The main difference was a constant shift in effective sound intensity, which was well predicted by a simple gain control scheme. We found expansive nonlinearities in low-frequency responses, which, with increasing frequency, gradually turned into the more familiar compressive nonlinearities. The overall power of distortion products was at least 13 dB below the overall power of the linear response, but in a limited band above the characteristic frequency, the power of distortion products often exceeded the linear response. Our results explain the partial success of a “quasilinear” description of wideband basilar membrane responses, but also indicate its limitations.

## Introduction

The cochlea performs a spectral analysis of sounds. Different frequency components excite different longitudinal locations of the cochlear partition. The nerve fibers innervating the cochlea thus become frequency channels, each fiber coding a limited band around its characteristic frequency (CF). Frequency selectivity precedes the transduction to neural signals, as shown by the sharp tuning of mechanical responses of the basilar membrane (BM). The cochlea also performs a dynamic-range compression, mapping a 100-dB range of stimulus levels onto a much smaller range of responses (Cooper 2004). Roughly speaking, each frequency band has its own dynamic compression. Even for elementary stimuli like single tones, the combination of spectral analysis and multi-channel dynamic compression gives rise to complex stimulus–response relations, which may alternatively be described as frequency-dependent growth or intensity-dependent filtering (Robles and Ruggero 2001).

For linear systems, the response to wideband stimuli can be constructed by summing pure-tone responses. This linear approach fails for strongly nonlinear systems like the cochlea. Several studies have reported frequency tuning of BM responses to wideband sounds: white noise (De Boer and Nuttall 2000, 2002; Recio-Spinoso et al. 2009), clicks (Recio et al. 1998; Recio and Rhode 2000), and tone complexes (Rhode and Recio 2001a, b). For all of these wideband stimuli, frequency tuning was found to be comparable to the single-tone case. The effects of sound pressure level (SPL) on tuning were similar, too: with increasing SPL, tuning became less sharp and shifted toward lower frequencies. The similarity in frequency tuning was analyzed in considerable detail by De Boer and Nuttall (2002). They compared amplitude–frequency curves between white noise and single-tone BM responses, and found the differences to be minimal—provided one is allowed to freely choose the SPLs of the stimulus pairs to be compared, thereby optimizing the match.

These findings suggest that only a modest alteration of the linear approach is needed to describe BM responses to stationary wideband stimuli. The idea is to employ a different linear filter for each SPL. This modified approach is known as intensity-dependent linear or “quasilinear” filtering (De Boer and Nuttall 2002; Recio-Spinoso et al. 2009), and it is closely related to the use of level-dependent auditory filters in psychoacoustic models (Lutfi and Patterson 1984). An explicit test of the quasilinear approach to white noise responses was provided by Recio-Spinoso et al. (2009), who estimated a linear filter from one set of white-noise BM responses and used it to successfully predict responses to independent noise stimuli having the same SPL.

The quasilinear approach becomes less straightforward when attempting to predict responses to one stimulus type (e.g., tones) from the responses of another stimulus type (e.g., noise). The similar frequency selectivity between responses to these two stimulus types suggests that quasilinearity is still a useful concept, but it is not obvious how to match the stimulus levels across stimulus types. De Boer and Nuttall (2002) tackled these questions using a specific cochlear model that incorporates saturating positive feedback by outer hair cells. Although several conclusions of that study may admit generalization beyond the model assumptions (see also De Boer 1997), it is important to analyze the relation between narrowband and wideband responses independently of specific cochlear models.

We report single-tone and wideband responses of the gerbil BM and address the following questions:

- Can wideband responses be predicted from single-tone responses (or vice versa)?
- What are the limitations of the quasilinear description?

Answering these questions required the isolation and scrutiny of the nonlinear aspects of BM responses, which yielded insights beyond the original questions.

Our stimuli were irregular tone complexes and we used Fourier analysis to study stimulus–response relations (Van der Heijden and Joris 2006; Meenderink and Van der Heijden 2011). This has several advantages over Wiener kernel analysis of noise responses. The signal-to-noise ratio is better owing to the concentration of stimulus power on a few discrete components, which allowed us to use true wideband stimuli, unlike the “composite responses” of De Boer and Nuttall (2002); the statistical significance of phase locking to the stimulus can be tested; odd order distortion products can be separated from linear stimulus components, which is not possible in the Wiener kernel approach (Johnson 1980).

## Materials and Methods

### Animal preparation

BM motion was measured from a single location in seven cochleae of Mongolian gerbils (*Meriones unguiculatus*; female, ~60 g), with CFs of 11.2–18.2 kHz. Methods were based on Cooper and Rhode (1992). All procedures were approved by the Erasmus MC laboratory animal committee.

Animals were anesthetized with intraperitoneal injections of ketamine/xylazine (initial dose, 80 and 12 μg/g body weight, respectively; subsequent doses, 1/3 of that each hour). A heating pad kept the body temperature at 37 °C. The head was glued to a head holder. A tracheotomy allowed the animal to breathe freely. The pinna of the left ear, its cartilaginous material, and the tissue slightly posterior from the ear canal were removed, exposing the bony rim of the ear canal and the superior mastoid chambers of the left bulla. Careful opening of the bulla with a scalpel, avoiding high intensity sounds, granted a clear view on the stapes, and BM through the round window. Middle ear muscles were left intact. After tearing the round window membrane using an insect pin, reflective beads (silver coated microspheres, 25 μm, 1.0–1.2 g/cm^3^ (hollow glass), or ~1.3 g/cm^3^ (PMMA); Nanoparticulate Surface Adhesion Ltd., Loanhead, UK) were introduced and allowed to settle on the BM. These beads have been shown to have negligible effect on BM motion (Cooper 1999). A glass cover slip overlying the round window area stabilized the air–fluid interface.

Experiments were carried out in a 45-min window within 2 h after tearing the round window, except experiment RG11349 where single-tone data were recorded 1 h after multitone data. All single-tone responses showed near-CF nonlinearities starting below 40 dB SPL, indicating a good cochlear condition (Ren and Nuttall 2001; Ren et al. 2011).

### Recording system

A personal computer running custom software in MATLAB (The MathWorks, Natick, MA, USA) calculated stimuli and sent them to a 24-bit D/A-channel (RX6; Tucker-Davis Technologies (TDT), Alachua, FL, USA) at 111.6 kHz. Signals were fed through a programmable attenuator (PA5; TDT) and an amplifier (SA1; TDT), and played over a speaker (CF1; TDT) connected to a custom-built sound delivery probe, which was sealed to the ear canal with Vaseline. Stimuli were compensated for the acoustic transfer of the sound delivery system using a cavity measurement and varied less than 4 dB in the 5–25 kHz range. Output spectra were monitored during the experiments using a probe microphone (40AG, G.R.A.S., Holte, Denmark).

A single-point laser vibrometer (OFV-534; Polytec, Waldbronn, Germany) with an illumination unit (VIB-A-510; Polytec), connected to a velocity decoder (VD-06; Polytec), measured a single bead’s vibrations through a 5× microscope objective (M Plan Apo 5×, NA = 0.14, *f* = 40; Mitutoyo, Veenendaal, The Netherlands). Decoder output was sampled by a 24-bit A/D-converter (RX6; TDT) at 111.6 kHz and stored on hard disk. A double-walled sound-proof booth (Acoustair, Moerkapelle, The Netherlands) and an optical table (Newport, Irvine, CA, USA) isolated the experiments from ambient noise.

### Stimuli

Each experiment started with measuring stapes velocity in response to a series of 80-dB SPL single tones in the 5–25-kHz range (700-ms duration including 2-ms rise/fall, presented each 1,000 ms; 0.01 octave steps; two repetitions). Stapes response was assumed linear; the reflex of the middle ear muscles has little effect above 5 kHz (Møller 1965; Rosowski et al. 2006). Stapes velocity, measured by pointing the laser beam to a reflective bead placed near the footplate, was used as a reference for the input to the cochlea. Thus, the phase data (Figs. 2 and 3) relate BM motion toward scala vestibuli to inward stapes motion.

Using the same stimulus protocol, single-tone BM responses were measured from 5 to 25 kHz in 0.05 octave steps at 0–80 dB SPL in 10-dB steps. The order of the tones was randomized. Amplitudes and phases of the responses were evaluated using Fourier analysis. CF was determined from the peak of the lowest SPL velocity–frequency curve, normalized to stapes motion.

The multitone stimuli consisted of 40 frequency components, irregularly spaced over 5–25 kHz, such that combination tones up to the third order never coincided with any of the 40 primary components (*f*_i_ ≠ *f*_j_, *f*_i_ ≠ *f*_j_ ± *f*_k_, *f*_i_ ≠ *f*_j_ ± *f*_k_ ± *f*_l_; see also Victor 1979; Van der Heijden and Joris 2006; Meenderink and Van der Heijden 2011). In order to avoid any spectral splatter in the Fourier analysis of the responses, the stimulus waveform must contain an integer number of cycles of each of the primary frequencies. Thus, all *f*_k_ must be integer multiples of a fundamental frequency, *f*_k_ = *f*_0_ *n*_k_. This makes the problem of finding the frequencies *f*_k_ equivalent to finding positive integers *n*_k_ obeying the same inequalities as the *f*_k_. This nontrivial numerical problem is akin to that of the “Golomb ruler” (Bloom and Golomb 1977). We solved it by “brute force”, i.e., by testing random sequences {*n*_k_}. The number of inequalities *N*_ineq_ grows rapidly with the number of primaries (as a third-order polynomial). For 40 components, *N*_ineq_ = 44,280, and it is unavoidable that sets of numbers {*n*_k_} simultaneously obeying these 44,280 inequalities contain large values; typically, max(*n*_k_) ≈ 125,000. Given the ~25-kHz range of our stimuli, this implies that the fundamental frequency *f*_0_ is in the order of 1/5 Hz, corresponding to a 5-s cycle.

Within each experiment, the frequencies and starting phases of the stimulus components were fixed, making the stimuli identical within one experiment. The multitone stimuli were varied across experiments. Duration of all multitone stimuli was three times its fundamental cycle of ~5 s, amounting to 15.05 s. The amplitude was equal for all stimulus components. Intensity was increased in 10-dB steps from 0 to 80 dB SPL per component. When playing the multitone stimulus at 80 dB SPL per tone, acoustic distortion, measured in a cavity using a ¼″ microphone (Brüel and Kjaer, Naerum, Denmark) and evaluated as described in the text addressing Figure 8, was ~46 dB below the stimulus components, independent of frequency in the 5–25 kHz range.

### Analysis

Response components were evaluated using Fourier analysis. For both single-tone and multitone responses, there is an intrinsic difficulty in estimating the noise floor at a given frequency. In BM recordings, this is particularly bothersome, because floor effects at low SPLs are easily confounded with compressive growth of the response. We therefore tested individual response components for their phase locking to the stimulus. Each 15-s response waveform was divided in 10 equally long, non-overlapping sections. To analyze a given section, the samples of the other sections were set to zero, preserving the total number of samples in the waveform. The section under investigation was windowed using 25-ms cosine-squared ramps and the phases were computed from an FFT of the entire waveform. For each spectral component, a Rayleigh test (*p* < 0.001) of its 10 phase values determined whether the data point was accepted.

For the calculation of explained variance of the phase predictions of the gain control model, we used the circular variance (Fisher 1993) defined as

$$ {{\sigma }^{2}} = 1 - \left| {\frac{1}{N}\sum\limits_{{k = 1}}^{N} {\exp \left( {i{{\varphi }_{k}}} \right)} } \right|, $$

where |.| denotes the absolute value.

## Results

### Rayleigh test

**FIG. 1:**
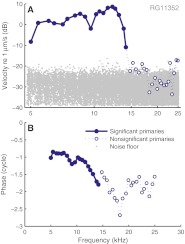
Using the Rayleigh test to determine significance of phase locking to stimulus components of analog recordings. *Solid lines* and *solid symbols* mark the subset of data meeting the *p <* 0.001 criterion; *dotted lines* and *open symbols* indicate failure to meet it. **A** and **B** BM amplitude and phase responses to a 20-dB SPL multitone stimulus.

Figure 1 shows the amplitude and phase of a multitone response. The Rayleigh test clearly distinguishes between significant responses (*solid circles*, above the noise floor) and nonsignificant responses (*open circles*, in the noise floor). The nonsignificant responses above 15 kHz show erratic phase behavior, consistent with random data. Insignificant components have been discarded from all data presented below.

### Isointensity curves

**FIG. 2:**
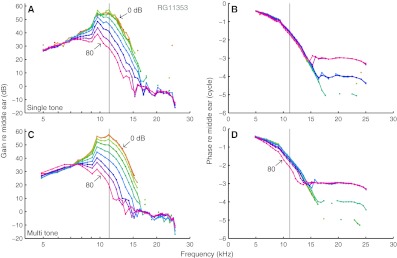
Isointensity curves. BM amplitude (**A**, **C**) and phase (**B**, **D**), expressed *re* stapes motion, in response to single-tone and multitone stimuli, respectively. *Vertical black lines* indicate CF. SPL per component was varied in 10-dB steps as indicated in the graph. CF = 11.2 kHz.

**FIG. 3:**
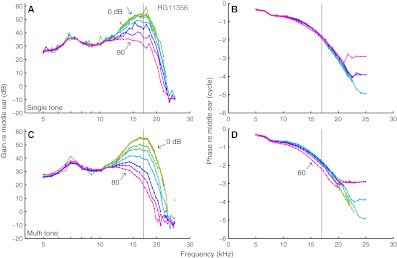
Layout as Figure 2. Different ear. CF = 17.0 kHz.

Normalized responses are shown in Figures 2 and 3 for two experiments (CF = 11.2 and 17.0 kHz). The shapes of the isointensity curves were very similar between single-tone data (upper panels) and multitone data (lower panels). Low-SPL responses were linear, as reflected by overlying curves. As SPL increased, both single-tone and multitone responses showed similar compressive nonlinear growth (<1 dB/dB) of components near CF, visible as a reduction of the normalized magnitude (panels A and C of Figs. 2 and 3). This reduction of sensitivity with increasing SPL was accompanied by small, but systematic phase changes (panels B and D of Figs. 2 and 3).

For frequencies well above CF, the responses showed a high-frequency plateau, reflected by a flat, SPL-independent gain and a constant phase approximately equal to the middle-ear phase. The plateau extended to lower frequencies with increasing SPL. Well below CF (<0.7 × CF), the multitone responses showed a slightly expansive nonlinear growth, visible as an increased sensitivity at high SPLs (>70 dB SPL). The irregularities in amplitude and phase around 10 kHz in Figure 2 originate from the middle-ear responses and probably reflect the transition from piston-like to rocking motion of the stapes (de La Rochefoucauld et al. 2008; Ravicz et al. 2008).

Compression started at lower SPLs for multitone stimuli than for single tones, and, for example, the 80-dB SPL multitone curves showed a larger reduction in sensitivity near CF than any of the single-tone curves. Since the intensity of the multitone stimuli is specified as SPL per tone, the response to individual components was clearly affected by the presence of other components. For the cochlea with its entangled compression and frequency selectivity, it is not a priori clear how to account for such across component influences. We start by quantifying the systematic differences between single-tone and multitone responses.

### Equivalent SPL

The similar shapes of single-tone and multitone curves (Figs. 2 and 3) suggest a description of the SPL dependence in terms of a single parameter, which we call “effective level.” By assumption, its value determines the responses to all frequency components, independent of whether they are presented consecutively or simultaneously. We aim at matching the SPLs of the two stimulus conditions (single tone and multitone) that correspond to the same effective level. Specifically, given a single-tone isointensity curve, we determined the SPL of best matching multitone response (“equivalent multitone SPL”).

**FIG. 4:**
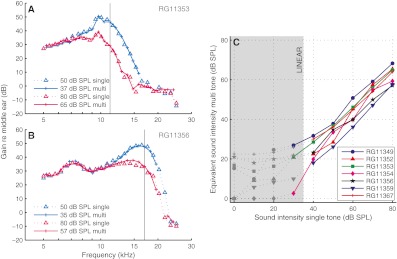
Similar frequency tuning of single-tone and multitone responses. **A**, **B** For two experiments, two isointensity single-tone responses and their equivalent-level multitone responses are shown. *Vertical black lines* mark CF. CF = 11.2 (**A**) and 17.0 (**B**). **C** Relation between single-tone SPL and equivalent multitone SPL for seven ears. The low SPL, linear range is marked by *gray markers*, *dotted lines*, and a *gray area*.

The multitone data were first interpolated to a fine grid of SPLs and frequencies. Next, minimization of the sum of squared differences led to the (interpolated) multitone curve that best matched the single-tone curve. Figures 4A, B show examples of single-tone curves together with the best matching multitone curves. These examples illustrate the good match in “filter shapes” between the single-tone and multitone data. The correlation between entire families of single-tone data (such as shown in Figs. 2A and 3A) and their matched multitone counterparts was 0.991 ± 0.005 (*N* = 7). This near equivalence in frequency tuning is a nontrivial observation in itself (see also De Boer and Nuttall 2002), which also justifies the parametric description of the level dependency in terms of equivalent SPL.

Figure 4C shows the relation between single-tone SPLs and their equivalent multitone SPLs for seven ears. The linearity of low-level (<30–40 dB SPL) single-tone responses causes them to match well with *any* of the low-level (<10–20 dB SPL) multitone responses. In this linear range (shaded area in Fig. 4C), the concept of an equivalent SPL became ambiguous. Outside the linear range, however, the correspondence was systematic. As expected from Figures 2 and 3, the equivalent multitone SPL grew monotonically with single-tone SPL, and the latter was higher than the former. Linear fits yielded slopes of 0.96 ± 0.11 (*N* = 7). Equivalent SPL is therefore well described by a uniform offset *Δ*:

1 $$ {\text{SP}}{{\text{L}}_{\text{multi}}} = {\text{SP}}{{\text{L}}_{\text{single}}} - \Delta $$

This offset, however, varied considerably across experiments (*Δ* = 5.7–25.1 dB; 16.4 ± 4.1 dB). Since the total power in the 40-tone complex is 16 dB above the SPL per tone, the fact that *Δ* often exceeded 16 dB indicates that across-component interaction (suppression) can exceed compression (“auto-suppression”) of single-tone responses. There was a positive correlation between CF and *Δ* (*R* = 0.863, *p* = 0.013). The fixed, 5-kHz low cutoff of our stimuli introduced a correlation between CF and the number of below-CF components, so part of the spread in *Δ* may come from the dominance of low-side suppression at high SPLs (low-side suppression grows steeply with SPL (Rhode and Cooper 1993; Cooper 1996; Rhode 2007b)). Alternatively, the correlation of CF and *Δ* may indicate that the amount of compression varied with CF. Our data do not allow a further analysis of the spread in *Δ*.

### Predicting Single-Tone from Multitone Responses

**FIG. 5:**
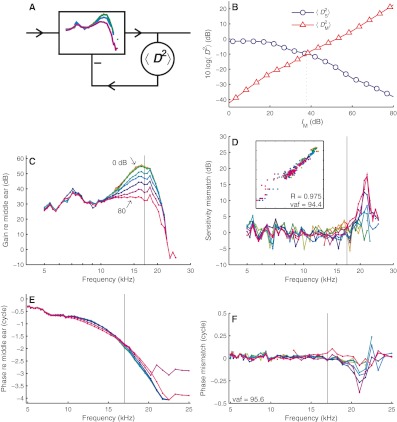
Predicting single-tone responses from multitone responses. **A** Automatic gain control model. The input passes a variable-gain filter that reproduces the measured multitone responses. The overall magnitude of the output, $\left\langle {{{D}^{2}}} \right\rangle$, is fed back to the filter as a gain control, setting the shape of the filter. **B** Predicting a single-tone response. Passing a 50-dB near-CF single tone to all possible filters produces $\left\langle {{D_{\text{S}}}^{{2}}} \right\rangle$ as a function of the multitone level *I*_M_ associated with the respective filters. For the filter setting to be self-consistent, $\left\langle {{D_{\text{S}}}^{{2}}} \right\rangle$ must match the $\left\langle {{D_{\text{M}}}^{{2}}} \right\rangle$ associated with the candidate filter. **C**, **E** Model predictions of the data in Figure 3 for normalized amplitude and phase, respectively. **D**, **F** Difference between predictions and data plotted versus frequency for amplitude and phase, respectively. Each curve represents a different SPL. *Inset* in **D** scatter plot of model predictions versus data for amplitude, with correlation *R* and variance accounted for (vaf) indicated. Variance accounted for (vaf) in phase indicated in **F**.

The similarity between single-tone and multitone sensitivity curves (Figs. 2 and 3) suggests that one can be predicted from the other. The matching of the responses (Fig. 4) depended on a single parameter, the effective level, which, however, did not have an obvious relation to the *stimulus* intensity. For instance, if it were equal to the total SPL of the stimulus, the effective levels of single-tone and multitone stimuli should differ by a fixed 16 dB. The alternative assumption, namely, that effective level depends on the local *response* magnitude, leads to an automatic gain control scheme as shown in Figure 5A. The collection of (interpolated) multitone sensitivity curves at various SPLs serves as the realizable filter shapes of a variable-gain filter. The gain setting of the filter is controlled by the magnitude of its own *output*, with higher output magnitudes invoking lower gains. In this scheme, the multitone sensitivity data play a double role: they provide both the collection of filter shapes and the association of output magnitude with individual shapes. For the output magnitude, we took the mean square BM displacement denoted $\left\langle {{{D}^{2}}} \right\rangle$. Note that the scheme does not involve any fitting parameters. The single-tone responses follow unambiguously from the multitone responses.

The predictions of the single-tone responses were obtained as follows. The amplitude and phase of the multitone responses (Figs. 3C, D) were cast into an SPL-dependent complex (stapes to BM) transfer function

2 $$ {H_{\text{BM}}}\left( {f,{I_{\text{M}}}} \right), $$

where *f* is the frequency and *I*_M_ is the SPL per tone of the multitone stimuli. This equation expresses the assumption that at each fixed value of *I*_M_ the filter is linear. Linear interpolation was used to estimate *H*_BM_ in between the measured grid of frequencies and intensities. The next step is to apply these transfer functions to single-tone stimuli. Denoting the middle ear transfer function (relating pressure in the ear canal to stapes motion) as

3 $$ {H_{\text{ME}}}(f), $$

the mean square displacement of BM vibration in response to a single tone of amplitude *A* (expressed *re* 20 μPa) and frequency *f* is predicted to be

4 $$ \left\langle {D_{\text{S}}^2} \right\rangle = \tfrac{1}{2}{\left| {{\text{A}}{{\text{H}}_{\text{ME}}}(f){H_{\text{BM}}}{{{\left( {f,{I_{\text{M}}}} \right)}} \left/ {{2\pi f}} \right.}} \right|^2}. $$

At this stage, it is not clear which value of the equivalent multitone level *I*_M_ should be applied to a given single-tone stimulus. The blue circles in Figure 5B show an example of $\left\langle {{D_{\text{S}}}^{{2}}} \right\rangle$ as a function of *I*_M_. It is a descending function because the single tone has fixed amplitude and is subjected to a gain that is negatively controlled by *I*_M_.

For a multitone stimulus presented at *I*_M_ dB SPL per tone, the amplitude of the tones is

5 $$ A = {10^{{{{{{I_{\text{M}}}}} \left/ {{20}} \right.}}}} $$

and the mean square displacement in response to this multitone stimulus adds up to

6 $$ \left\langle {D_{\text{M}}^2} \right\rangle = \tfrac{1}{2}\sum\limits_{{k = 1}}^{{40}} {{{\left| {{{10}^{{{{{{I_{\text{M}}}}} \left/ {{20}} \right.}}}}{H_{\text{ME}}}\left( {{f_k}} \right){H_{\text{BM}}}{{{\left( {{f_k},{I_{\text{M}}}} \right)}} \left/ {{2\pi {f_k}}} \right.}} \right|}^2}} . $$

The red triangles in Figure 5B show $\left\langle {{D_{\text{M}}}^{{2}}} \right\rangle$ as a function of *I*_M_. It is an ascending function because BM displacement grows with the SPL of a multitone stimulus. The true control parameter in the feedback scheme is the mean square displacement of the BM. (*I*_M_ merely serves to label the control parameter in a way that provides a direct link to the multitone data.) Therefore, the correct choice of *I*_M_ in the single-tone case (or any stationary stimulus) is that value that, when applied to the stimulus, yields the same mean square BM displacement as a multitone stimulus presented at *I*_M_ decibels. In formulas, one has to find the *I*_M_ for which $\left\langle {{D_{\text{S}}}^{{2}}\left( {{I_{\text{M}}}} \right)} \right\rangle = \left\langle {{D_{\text{M}}}^{{2}}\left( {{I_{\text{M}}}} \right)} \right\rangle$. This amounts to determining the intersection of the two curves in Figure 5B. For each single tone having frequency *f* and sound level *L*, this procedure yielded an equivalent multitone level *I*_M_(*f*,*L*). The multitone transfer functions *H*_BM_(*f*, *I*_M_(*f*,*L*)) then provided the prediction of the response amplitude and phase for that single tone. The resulting predictions are shown in Figures 5C, E.

Figure 5D compares measured and predicted single-tone normalized amplitudes. For this ear, the predictions explained 94.4 % of the variance. For the seven ears tested, the average correlation between data and predictions was *R* = 0.955 ± 0.037; the explained variance, 91.2 ± 6.9 %. The largest prediction errors occurred above CF, where automatic gain control overestimated high-SPL response magnitudes. Figure 5F compares measured and predicted phases. Using the circular variance metric (see Materials and Methods section), the explained variance of the phase data of this ear was 95.6 %. For the seven ears tested, the explained variance was 91.8 ± 3.4 %. Again, the largest deviations occurred above CF at the highest SPLs, where the gain control scheme predicted too much phase lag. An alternative approach, in which the filter was controlled by BM velocity (rather than displacement) yielded only minimal (0.12 ± 0.23 dB) changes in the predictions; the limited frequency span of our data prevents a critical comparison between the methods. Our choice for BM displacement was motivated by its consistency as a predictor of suppression (Fig. 2 of Cooper 1996).

### SPL-dependent changes in sensitivity and phase

**FIG. 6:**
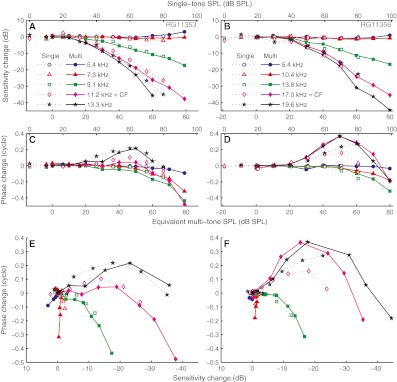
SPL-induced sensitivity and phase changes. Columns show two different experiments. *Dashed* and *solid lines* indicate single-tone and multitone responses, respectively. **A–D** For a subset of frequencies (indicated in the graphs), magnitude and phase data are shown after subtraction of their low-SPL, linear values. *Upper* and *lower abscissae* indicate the SPL per component of the single-tone and multitone stimuli, respectively. Their offset was based on the matching of the effective levels (Fig. 4C). **E** The data of **A** and **C** with phase change plotted directly against sensitivity change. The *inverted abscissa* emphasizes the reduction of sensitivity with growing SPL. **F** Layout as panel **E**; data from panels **B** and **D**.

Figure 6 shows the effects of SPL on sensitivity and phase, expressed as changes *re* the low-SPL (linear) responses. The use of a low-SPL reference highlights and quantifies any deviations from linearity and facilitates the comparison with suppression data (Cooper 1996). The sensitivity graphs may be viewed as input/output curves with a 1 dB/dB reference subtracted. Linear growth yields horizontal lines, and expansive and compressive growth yield positive and negative slopes, respectively. Single-tone and multitone data are shown together, but horizontally shifted to match their effective SPLs. Well below CF, both single-tone and multitone responses showed little change in sensitivity or phase for SPLs up to ~70 dB SPL. The high-SPL multitone stimuli, however, showed an expansive growth (Figs. 6A–D, *blue curves*). For somewhat higher frequencies, a slight phase lag occurred at the highest SPLs, without any change in sensitivity (Figs. 6A–D, *red triangles*). For frequencies closer to (but still well below) CF, sensitivity started to drop at moderate SPLs, while phase started lagging at high (>70 dB) SPLs only (Figs. 6A–D, *green squares*). Near CF, sensitivity started to decrease at even lower SPLs, and dropped steeply with SPL. Phase varied nonmonotonically; the mid-SPL lead diminished with SPL and turned into a lag at high SPLs (Figs. 6A–D, *magenta diamonds*). Finally, above CF (yet below the plateau, which is not considered), the largest reductions in sensitivity occurred, accompanied by phase leads exceeding those at CF. Again, phase leads decreased at the highest SPLs (Figs. 6A–D, *black stars*).

After matching effective levels, the SPL dependence of sensitivity became similar between single-tone and multitone responses (Figs. 6A, B, *dashed lines with open symbols* versus *solid lines with solid symbols*). The phase changes of the single-tone and multitone responses also shared the same trends, but the match was less perfect (Figs. 6C, D).

An alternative way of visualizing the nonlinear effects of SPL is to plot phase change directly against sensitivity change (cf. Cooper 1996, Fig. 5). This removes effective SPL from the graph and allows a direct comparison of single-tone and multitone responses. Each frequency is now represented by a curve (Figs. 6E, F) that starts in the origin (zero change in sensitivity and phase) and moves away from it with increasing SPL. The effects described for Figures 6A–D are now seen as a lengthening of the curves and simultaneous counter-clockwise rotation around the origin with increasing frequency. Although the single-tone and multitone responses showed some differences, the overall pattern was the same.

**FIG. 7:**
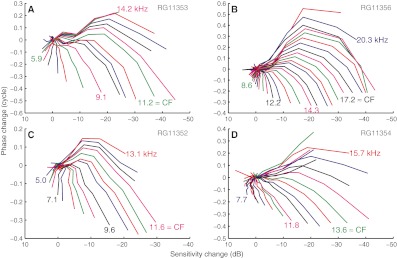
Phase change against sensitivity change for all frequencies of multitone recordings. *Colored labels* indicate the frequencies (kHz) of the closest curve with the same color. Panels contain data from different ears.

The rotation effect was systematic and robust. Figure 7 shows phase change versus sensitivity change for all 40 frequency components of the multitone responses. All four ears show the peculiar nonlinear effect, mentioned above, of an initial expansive nonlinearity with a slight SPL-induced phase lag (Fig. 7C, *blue 5.0 kHz*) that gradually changes to a larger phase lag without a sensitivity change (Fig. 7C, *black 7.1 kHz*). At still higher frequencies, the nonlinearity adopts the more familiar compressive character. Importantly, neither the expansive nonlinearity nor the “phase-only” nonlinearity is an isolated phenomenon. They are merely stages in a gradually transforming nonlinear pattern.

### The Limits of Quasilinearity: Distortion Products

In Recio-Spinoso et al. (2009), first-order Wiener kernels obtained from white-noise BM responses, were shown to successfully predict BM responses to independent, same-SPL noise waveforms. Because different SPLs required different linear descriptions, they described their findings as “quasi-linear filtering.” This raises the question how linear the cochlear responses to wideband stimuli really are. Two-tone stimuli can produce audible and measurable odd order distortion product (DPs). Does wideband stimulation somehow “linearize” the cochlear response or is the amount of distortion comparable to the two-tone case? Such questions cannot be answered by an analysis of white noise responses (Johnson 1980). The multitone stimuli of the present study, on the other hand, allow an analysis of the third-order DPs (DP3s), since they never coincide with the primaries (see Materials and Methods section).

**FIG. 8:**
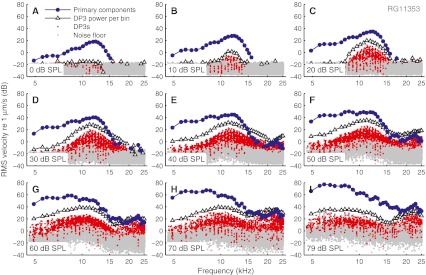
Linear response components and distortions. All panels show power spectra of multitone responses. Phase-locking primary components are marked by *blue circles*; and phase-locking DP3s by *red dots*. *Black triangles* indicate the power of DP3s summed over the frequency band around each primary. *Gray dots* are the remaining components. Each panel shows data for a given SPL per component as indicated in the graphs. CF = 11.2 kHz.

Figure 8 shows power spectra of multitone responses obtained at various SPLs. Blue solid circles mark the primary components; red dots mark DP3s that were significantly phase locked to the stimulus (Rayleigh test, *p* < 0.001). In most cases, the DP3s outnumbered the primary components. To evaluate the relative levels of DP3s and linear response, we summed the power of all the DP3s falling in each frequency band around a primary; the geometric means between subsequent primaries served as edges of bins. The summed power of the DP3s in each bin (*P*_DP3_) is shown as triangles in Figure 8. It is to be compared with the power of the linear response components at the same frequency (*P*_LIN_).

This comparison revealed a number of systematic features. At the lowest SPLs, no significant DP3s were observed. With increasing SPL, DP3s began to appear, and their power *P*_DP3_ grew faster than *P*_LIN_. Often, the appearance of DP3s at the lowest SPLs was a more sensitive indicator of the emergence of nonlinearity than the reduction of sensitivity (e.g., compare Figs. 8B and 2C). At higher SPLs, the growth of DP3s slowed down and fell behind that of *P*_LIN_.

Compared to the linear response, the DP3s showed a bias towards higher frequencies, reflected by a shallower high-frequency roll-off (Fig. 8). As a result, the ratio *P*_DP3_/*P*_LIN_ depended strongly on frequency for all SPLs. Below CF, *P*_DP3_ was typically 20 dB or more below *P*_LIN_. Near CF, *P*_DP3_ came as close as 10 dB below *P*_LIN_ (ear RG11359 came as close as 6 dB). Above CF, *P*_DP3_ often even exceeded *P*_LIN_ (Figs. 8D–F) indicating a very strong degree of distortion at intermediate SPLs.

**FIG. 9:**
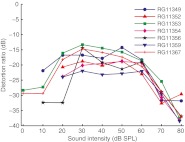
Distortion ratio versus SPL extracted from the responses to multitone stimuli for seven ears. This ratio compares the power of third-order DPs, summed over the entire frequency range, with the summed power of the linear response components. Based on the number of DPs available (*N* = 21,274) and the Rayleigh criterion (*p <* 0.001) we required a minimum of 2 *Np* = 43 significant DPs for a data point to be included.

The overall degree of distortion (disregarding frequency) was assessed by summing the power of both the linear response components and the DP3s over all frequencies. The resulting “distortion ratio” Σ*P*_DP3_/Σ*P*_LIN_ (shown for seven ears in Fig. 9) peaked at intermediate SPLs, but never exceeded −13 dB, consistent with a quasilinear characterization of BM responses.

## Discussion

### Rayleigh test and low-SPL linearity

The Rayleigh test is routinely used to assess significance of phase locking of neural spike trains to tones. We showed (Fig. 1) that it is applicable to analog data too, providing a straightforward way to judge whether a Fourier component reflects a phase-locked response to the stimulus or is part of the noise floor. The test is generally applicable to the analysis of responses to periodic stimuli, e.g., evoked otoacoustic emissions. In cochlear mechanics, it distinguishes low-SPL floor effects from genuine compressive growth. After restricting the analysis to significant responses, we found linear growth at the lowest SPLs (Figs. 2 and 3) for all frequencies, in agreement with previous studies of BM responses in gerbil (Cooper 1999; Ren and Nuttall 2001).

### Comparison between wideband and tone responses

Previous studies (clicks (Recio et al. 1998), noise (De Boer and Nuttall 2000, 2002; Recio-Spinoso et al. 2009), and multitones (Rhode and Recio 2001a, b)) have shown that wideband responses share general characteristics with single-tone responses: compressive growth, widening and downward shifts of frequency tuning with SPL. The present study confirmed this similarity, analyzed it in greater quantitative detail, and explored its limits.

For each of the seven ears, the set of isointensity response curves obtained with tone complexes was very similar to the set of single-tone curves (Figs. 2 and 3). The individual curves matched well across the two sets when accounting for a fixed difference *Δ* in effective SPL between the multitone and single-tone data (Fig. 4). *Δ* varied considerably across experiments, but could be predicted in each case from the wideband responses by a simple gain control scheme without introducing any free parameters (Fig. 5).

Apart from the implications for cochlear mechanisms, our findings may inspire heuristic models of the auditory periphery. For instance, the effects of SPL on frequency resolution have long been known from behavioral data on auditory masking (Wegel and Lane 1924). Attempts to explain such data in terms of level-dependent filter shapes have raised the question how to incorporate stimulus intensity (Lutfi and Patterson 1984). The success of our gain control scheme suggests that the correct approach is to use a negative feedback configuration in which the filter is controlled by the magnitude of its own output, as previously proposed by Rosen and Baker (1994) and Lyon (2011).

### Comparison with De Boer and Nuttall (2002)

De Boer and Nuttall compared frequency selectivity of BM responses to single tones and noise bands, and analyzed their findings using a cochlear model that incorporated a saturating feedback from outer hair cells. Their amplitude–frequency curves (no phase data were presented) led them to conclude that “the response of the cochlea to [single] tones is, to a good approximation, equivalent to that of components of a noise signal” and that “insofar as frequency filtering […] is concerned, the cochlea is not ‘more nonlinear’ or ‘less nonlinear’ for [single] tones than for noise.” Our analyses of amplitude and phase curves (Figs. 2, 3 and 4), SPL dependence of sensitivity and phase (Figs. 6 and 7), and DP spectra (Fig. 8) confirm and generalize their conclusions. On the issue of equivalent levels, De Boer and Nuttall remarked that “stimuli of the appropriate levels have to be compared.” Interestingly, the offset between equivalent single-tone and wideband levels, which in our case varied over a ~20 dB range, appears to be less variable in their study (their Figs. 3, 4, 5, and 6 suggest a ~5-dB range). This may well be caused by the following methodological difference. The limited dynamic range of the reverse correlation analysis prevented De Boer and Nuttall from using true wideband stimuli. Instead, their amplitude–frequency curves were constructed by combining separate responses to single-octave noise bands. The authors justified using such composite curves by noting that “[in] the lower frequency ranges the cochlea is (nearly) linear.” This, however, ignores the strongly suppressive effect that low-frequency components have on the response to near-CF components. The absence of low-frequency components in De Boer and Nuttall’s CF-centered stimuli has probably weakened the effects of low-side suppression—particularly at high SPLs, because low-side suppression grows steeply with SPL. The larger bandwidth of our stimuli favored the role of low-side suppression, and (as discussed in connection with Fig. 4) this may well account for the larger variability in the offset between equivalent levels.

### SPL-induced changes of sensitivity and response phase

In using low-SPL, linear BM responses as a reference for describing nonlinear amplitude and phase (Figs. 6 and 7) we have followed Cooper (1996), who introduced it to quantitatively compare single-tone nonlinearity (compression) with across-component nonlinearity (suppression). Cooper’s findings indicated a common origin of compression and suppression, and laid out the foundation for a unified description of cochlear nonlinearity in terms of the effects of SPL on sensitivity and phase. In several aspects, the present study is an extension of Cooper’s. The use of multitone stimuli allowed us to extend the “probe” (suppressed tone) to a much larger frequency range than the near-CF tones used previously.

Our findings confirm and generalize those of Cooper: compression and suppression are well described by the same gradual reduction of sensitivity with SPL; the nonlinear phase effects are also consistent between the two cases. In this context, our gain control scheme (Fig. 5) may be viewed as a generalization of Cooper’s analysis (Fig. 2 of Cooper 1996) of the dependence of sensitivity on the total magnitude of BM displacement. The weakness of the scheme, i.e., the underestimation of suppression (*c.q.* sensitivity reduction) of above-CF tones, is a common finding of both studies.

The gain control scheme is based on the simplification that frequency tuning of excitation and suppression is identical. Unsurprisingly, the scheme performs worst exactly there where this assumption fails: just above CF, where suppression is stronger than expected from the steep high-frequency flanks of excitation (Schmiedt 1982). The mismatch is probably caused by the spatially distributed character of cochlear nonlinearity. Traveling waves of (above-)CF probes suffer most of their reduction at locations basal to the recording site, where any above-CF “suppressor” (including the tone itself in the single tone case) has a larger magnitude than at the recording site. Following the same line of reasoning, De Boer and Nuttall (2002) modified their cochlear model to allow the nonlinear feedback of outer hair cells to vary with cochlear location, which enabled them to account for their single-tone responses.

### Expansive nonlinearities below CF

High-SPL multitone responses showed a small but consistent expansive nonlinearity well below CF (Figs. 6 and 7). The absence of this effect in the single-tone data is likely due to their lower effective SPL (Fig. 4C). The expansive growth reported here differs from the expansion found in the apex, which occurred above CF (Zinn et al. 2000), but appears to match the below-CF, high-SPL expansive nonlinearities reported for gerbil (Cooper 1999) and chinchilla (Rhode 2007a), which have received little attention. The joint analysis of SPL-induced sensitivity and phase effects (Figs. 6 and 7) revealed that it is not an isolated effect. In fact, with increasing frequency there is a gradual transition from expansion to compression. (The smooth transition contrasts sharply with the violent return to linearity *above* CF that has all the marks of interfering response components; see Rhode (2007a).) An interesting type of nonlinearity occurs at the transition (~0.7 × CF) from expansion to compression: the response amplitude grows linearly with stimulus intensity, while the phase acquires a lag.

Invoking a qualitative version of “scaling invariance” (a postulated tradeoff between varying stimulus frequency and moving along the BM), the families of phase-sensitivity curves (Figs. 6E, F and 7) depict the progression of nonlinearity of a traveling wave as it propagates from base to apex. The lowest frequencies are identified with the most basal portion of the wave. Surprisingly, the SPL dependence in the initial nonlinear stage (basal portion of the wave) is expansive rather than compressive. As the wave moves on, the expansive nonlinearity is gradually reduced and turned into compression. Over that same trajectory, larger SPLs cause larger phase lags. This implies that the basal portion of the wave slows down when the SPL is increased, in agreement with experimental data (Ren et al. 2011). The combination of these three effects of increasing SPL: (1) initial increase in gain; (2) accelerated progressive decay of amplitude; (3) reduction of wave speed; suggests that higher SPLs cause the cochlear partition to become more compliant and more resistive at the same time, as proposed in Allen (1980).

### Distortion and the limits of quasilinearity

Wideband stimulation does not appear to alter the nonlinear character of BM responses. Wideband responses grew as compressively with stimulus amplitude as did single-tone responses, and showed the same SPL-induced changes in frequency tuning and response phase; the relative power of DPs (Fig. 8) was comparable to that observed with two-tone stimuli (Robles et al. 1997; Ren 2004). For moderate SPLs, the DP power density even exceeded that of linear response components in a limited band above CF (Figs. 8D–F). The occurrence of odd-order DPs at low SPLs was a very sensitive probe of the onset of compression (Fig. 8B). Despite all of these strongly nonlinear aspects, the linear components always dominated the *total power* of the wideband responses (Fig. 9). This global dominance of the linear response explains the success of quasilinear predictions of BM responses to white noise (De Boer and Nuttall 2000; Recio-Spinoso et al. 2009). Even in the mid-intensity range, the distortion ratio never exceeded −13 dB (Fig. 9), so at most 5 % of the response power originated from DPs. Thus, at least 95 % of the variance of the waveform was explained by the best linear model. The lower fractions (~90 %) of explained variance reported in Recio-Spinoso et al. (2009) may reflect a higher degree of nonlinearity in chinchilla than in gerbil and/or a suboptimal linear model.

## Conclusions

Expansive nonlinearities in the “low-frequency tail” of isointensity curves are not an isolated phenomenon, but represent one end of a systematic, gradual transformation of cochlear nonlinearity with frequency and place. The other, more familiar end, is the compression in the peak region of the traveling wave.

Single-tone and wideband BM responses are very similar in terms of frequency tuning and its dependence on SPL, compressive nonlinearity, and SPL-induced phase changes. The difference in their effective levels can be accounted for by local negative feedback without introducing any additional parameters. The largest mismatch between narrowband and wideband responses occurred just above CF, and our analysis suggests that this mismatch is caused by the same nonlocal aspects of cochlear nonlinearity that underlie differences in frequency tuning between excitation and suppression. The magnitude of third-order DPs evoked by wideband stimulation is comparable to DP magnitude observed in customary two-tone paradigms. The DP ratio (total power of DPs *re* total power of linear response) peaked at moderate (~40 dB SPL) levels, but never exceeded −13 dB, which explains the success of quasilinear descriptions of peripheral frequency selectivity. In a restricted band above CF, however, the power of DPs easily exceeded that of the linear response components. Stimulus bandwidth by itself has little effect on the strongly nonlinear character of the cochlear response.
